# Alterations of sensorimotor predictive processes and their electrophysiological signatures in Tourette syndrome

**DOI:** 10.1093/braincomms/fcaf458

**Published:** 2025-11-21

**Authors:** Thomas Schüller, Paola Mengotti, Adam Zabicki, Daniel Huys, Michael T Barbe, Gereon R Fink, Veerle Visser-Vandewalle, Simone Vossel, Juan Carlos Baldermann

**Affiliations:** Department of Neurology, University of Cologne, Faculty of Medicine and University Hospital Cologne, Cologne 50935, Germany; Department of Psychiatry and Psychotherapy, University of Cologne, Faculty of Medicine and University Hospital Cologne, Cologne 50935, Germany; Cognitive Neuroscience, Institute of Neuroscience & Medicine (INM-3), Forschungszentrum Jülich, Jülich 52425, Germany; Cognitive Neuroscience, Institute of Neuroscience & Medicine (INM-3), Forschungszentrum Jülich, Jülich 52425, Germany; Department of Psychology, Faculty of Human Sciences, University of Cologne, Cologne 50923, Germany; Department of Psychiatry and Psychotherapy, University of Cologne, Faculty of Medicine and University Hospital Cologne, Cologne 50935, Germany; Department of Psychiatry and Psychotherapy III, LVR Klinik Bonn, Bonn 53111, Germany; Department of Neurology, University of Cologne, Faculty of Medicine and University Hospital Cologne, Cologne 50935, Germany; Department of Neurology, University of Cologne, Faculty of Medicine and University Hospital Cologne, Cologne 50935, Germany; Cognitive Neuroscience, Institute of Neuroscience & Medicine (INM-3), Forschungszentrum Jülich, Jülich 52425, Germany; Department of Stereotactic and Functional Surgery, University of Cologne, Faculty of Medicine and University Hospital Cologne, Cologne 50935, Germany; Cognitive Neuroscience, Institute of Neuroscience & Medicine (INM-3), Forschungszentrum Jülich, Jülich 52425, Germany; Department of Psychology, Faculty of Human Sciences, University of Cologne, Cologne 50923, Germany; Department of Neurology, University of Cologne, Faculty of Medicine and University Hospital Cologne, Cologne 50935, Germany; Department of Psychiatry and Psychotherapy, University of Freiburg, Faculty of Medicine and University Hospital Cologne, Freiburg 79104, Germany

**Keywords:** Tourette syndrome, P300, Bayesian observer model, motor preparation, cue predictability

## Abstract

People with Tourette syndrome exhibit excessive motor actions known as tics. An aversive sensation called the premonitory urge often precedes these tics, leading to the conceptualization of Tourette syndrome as a sensorimotor disorder. In typical individuals, motor actions adapt flexibly to changes in the predictability of sensory cues. However, it remains unclear whether such sensorimotor predictions are altered in Tourette syndrome and, if so, which neural processes might underlie these changes. This study examined 30 individuals with Tourette syndrome and 30 control participants while recording EEG. Participants performed a motor cueing version of the Posner task, requiring behavioural adjustments to varying levels of cue predictability. Notably, while control participants exhibited the expected interaction between validity and cue predictability on motor responses, this effect was absent in individuals with Tourette syndrome. Neural signatures of flexible predictability-dependent processing were characterized by applying a Bayesian observer model to estimate trial-wise subjective beliefs about cue predictability from response speed and using these model-derived cue predictability estimates in single-trial regression analyses with EEG data. Our findings revealed that model-derived cue predictability modulated P3a amplitude, P3b onset and P3b amplitude differentially. Importantly, P3b amplitude modulations reflected beliefs about cue predictability, which were diminished in participants with Tourette syndrome. Overall, our results indicate that individuals with Tourette syndrome exhibit abnormal behavioural adaptation to the changing predictability of motor cues, suggesting an impaired processing of sensorimotor predictions. At the neural level, this is reflected by impaired activity associated with updating stimulus–response associations.

## Teaser

Schüller *et al*. found that individuals with Tourette syndrome show impaired behavioural and neural adaptation to changing cue predictability during a motor cueing Posner task. EEG and Bayesian modelling revealed reduced P3b-related processing of estimated beliefs about cue predictability, suggesting impaired processing of sensorimotor predictions in Tourette syndrome.

## Graphical Abstract

**Graphical Abstract:**
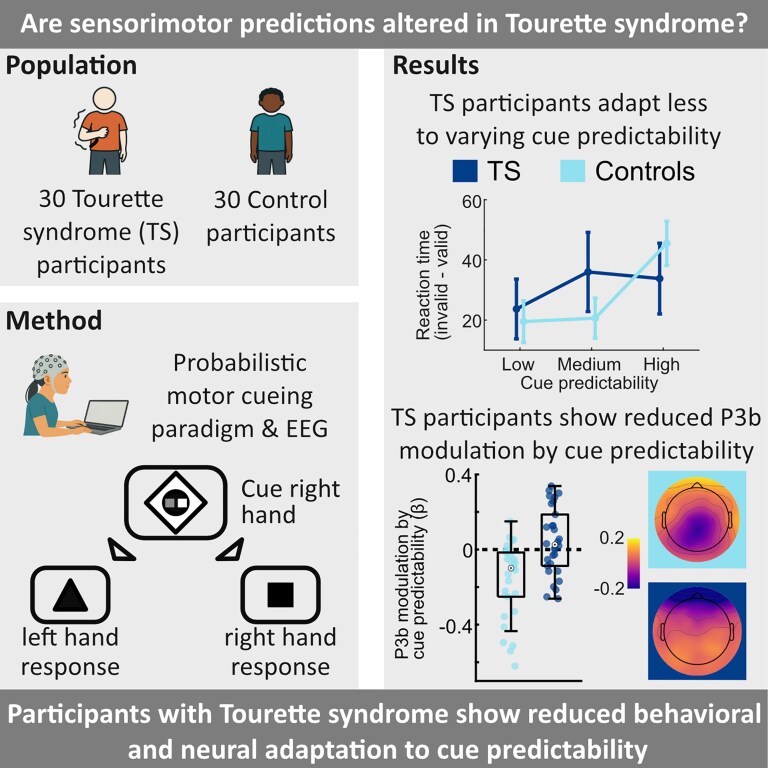

## Introduction

Tourette syndrome (TS) is a hyperkinetic neuropsychiatric disorder characterized by the chronic occurrence of tics—sudden, non-rhythmic, repetitive movements and vocalizations. A distressing sensation, the premonitory urge, commonly precedes the tics, which subsides following tic expression.^1,2^ The neuropsychological and neural mechanisms underlying the excessive emergence of urges and tics are still insufficiently understood. Evidence implicates a hyperdopaminergic state^3,4^ and functional disturbances in cortico-basal ganglia-thalamo-cortical networks^5-7^ in TS. Specifically, networks involved in sensorimotor processing and cognitive control may be critically affected.^8-10^ These networks play a vital role in cognitive processes, enabling flexible and adaptive responses in dynamically changing environments.^11,12^ The predictive coding framework provides a conceptual framework for explaining how expectations about the environment guide behaviour and are updated based on new observations. Specifically, when sensory input deviates from prior expectations, prediction error signals update those predictions to minimize future prediction errors and enable response adaptation.^13,14^ Findings have shown that TS may impair the adjustment of choices to changing statistical regularities, in line with deficient predictive coding mechanisms.^15-17^ A complementary line of research has focused on aberrant sensorimotor integration processes in TS, as evident in the strong interrelation between premonitory urges and tics. Rooted in the theory of event coding, the aberrant sensorimotor integration processes in TS are explained by the increased binding of stimulus features and actions in common ‘event files’.^18,19^ This idea received empirical support, showing attenuated sensory feature integration and increased event file bindings in TS.^19-22^

Posner-type cueing paradigms,^23^ in which a cue predicts the appearance of a target with a certain probability, have been used together with EEG to identify the neural processes underlying predictability-dependent behavioural adjustments. These studies have shown a modulation of event-related potentials by cue predictability, including the frontal–central P3a and the parietal P3b.^24,25^ Moreover, Bayesian modelling has demonstrated its capability to identify distinct processes important for behavioural adaptation to predictability, specifically associating P3a and P3b modulations with surprise.^26,27^ This closely aligns with foundational theories regarding P3 functions, where P3a is assumed to signify the redirection of attentional resources to unexpected events, while P3b modulations have been interpreted as context updating or, more specifically, the re-activation of stimulus–response mappings, which are both expected to scale with predictability.^28,29^ Furthermore, P3b onset latencies are related to reaction times (RTs) and, therefore, may reflect motor preparation processes.^30-32^ Preliminary studies with TS participants employed a Posner-like vibrotactile task with constant rates of cue predictability that led to either impaired^33^ or unaffected behavioural adaptations.^34^

Here, we employed a motor cueing version of the Posner task, adapted from Kuhns *et al*.,^35^ in TS participants and matched controls while recording EEG. The task was specifically designed to investigate predictability-dependent motor control, using a cue that probabilistically predicted an upcoming target, prompting preparation of the corresponding motor response. In contrast to previous studies,^24-26,35^ the targets in our study were presented centrally so that motor processes were not conflated with lateralized spatial orienting of attention. Crucially, the predictability of the cue (i.e. the proportion of validly and invalidly cued targets) varied throughout the experiment, prompting behavioural adaptation to this changing (i.e. volatile) predictability. When cue predictability is high, responses to validly cued targets are likely to be faster, whereas reactions to invalidly cued targets are likely to be slower, reflecting behavioural adaptation.^23,36^ To estimate trial-wise beliefs about cue predictability from each participant’s response speed (RS), we applied a Bayesian observer model [Hierarchical Gaussian Filter (HGF)^37,38^], as in previous work.^39^ In this study, this allowed for single-trial regression of model-derived (inferred) cue predictability with the EEG in TS participants and controls.

In summary, we systematically manipulated cue predictability to investigate its impact on behaviour, HGF model parameters and associated event-related potentials. If a maladaptation to volatile sensorimotor predictions (i.e. changing cue predictability) contributes to TS, this should be reflected in an attenuated modulation of cueing effects, reduced HGF learning parameters and altered modulations of P3a/b responses to inferred cue predictability.

## Materials and methods

### Participants

The study was performed in accordance with the Declaration of Helsinki and Good Clinical Practice guidelines, approved by the local ethics committee of the University of Cologne (Nr. 20-1443-2) and registered in the German Clinical Trials Register (DRKS00024834). All participants gave informed consent. Thirty-one control and 30 TS participants took part in the study. One healthy participant was excluded from the analysis due to recording problems. Therefore, the data of 30 control and 30 TS participants were analysed (see Table 1). *A priori* power analyses indicated that a sample size of 30 participants per group was needed to detect a medium effect size in group differences with 80% power. The control participants were matched with the TS participants for gender, age and education. Two TS participants and one healthy participant were left-handed. Five TS participants were treated with anti-dopaminergic medication, three with serotonergic medication and three with a combination of anti-dopaminergic and serotonergic medication. Three TS participants were treated with cannabinoids; one with a combination of anti-dopaminergic and cannabinoid medication; one with anti-dopaminergic, serotonergic and cannabinoid medication; and one with anti-convulsive medication. Thirteen TS and all control participants were not taking any medication at the time of the study. All participants had normal or corrected-to-normal vision. Control participants were not affected by any neurological or psychiatric disorder. To characterize the clinical profiles of our participants, we administered the following instruments: the Yale Global Tic Severity Scale^40^ (YGTSS) to quantify tic severity; the Premonitory Urge for Tics Scale^41^ (PUTS) to evaluate premonitory sensations; the Obsessive–Compulsive Inventory-Revised^42^ (OCI-R) for obsessive–compulsive symptomatology; the Wender Utah Rating Scale^43^ (WURS-K) for attention-deficit/hyperactivity symptoms; and the Beck Depression Inventory-II^44^ (BDI-II) for depressive symptomatology.

**Table 1:** Demographic and clinical data of Tourette and control participants

	Tourette participants	Control participants	*t*	df	*P*
Age	35.6 ± 10.6	37.2 ± 13.7	−0.49	58	0.62
Sex (M/F)	22/8	22/8	-	-	-
Handedness (R/L)	28/2	29/1	1.07	1	0.3
Years of education	11.5 ± 1.2	11.9 ± 1.3	−0.93	58	0.35
BDI-II	13.3 ± 11.8	3.2 ± 3.2	4.5	58	<0.001
WURS-K	25.6 ± 14.5	12.2 ± 10.7	4.01	58	<0.001
OCI-R	20.4 ± 15.2	9.6 ± 7.2	3.43	58	0.001
PUTS	37.8 ± 10.8	-	-	-	-
YGTSS-Global	45.5 ± 20	-	-	-	-
YGTSS-Tic	22.3 ± 8.7	-	-	-	-

Data are presented as means ± standard deviations. *P*-values refer to two-sample *t*-tests or *χ*^2^ for handedness.

BDI-II, Beck Depression Inventory II; WURS-K, Wender Utah Rating Scale; OCI-R, Obsessive–Compulsive Inventory-Revised; PUTS, Premonitory Urge for Tics Scale; YGTSS, Yale Global Tic Severity Scale.

### Stimuli and experimental paradigm

**Figure 1:**
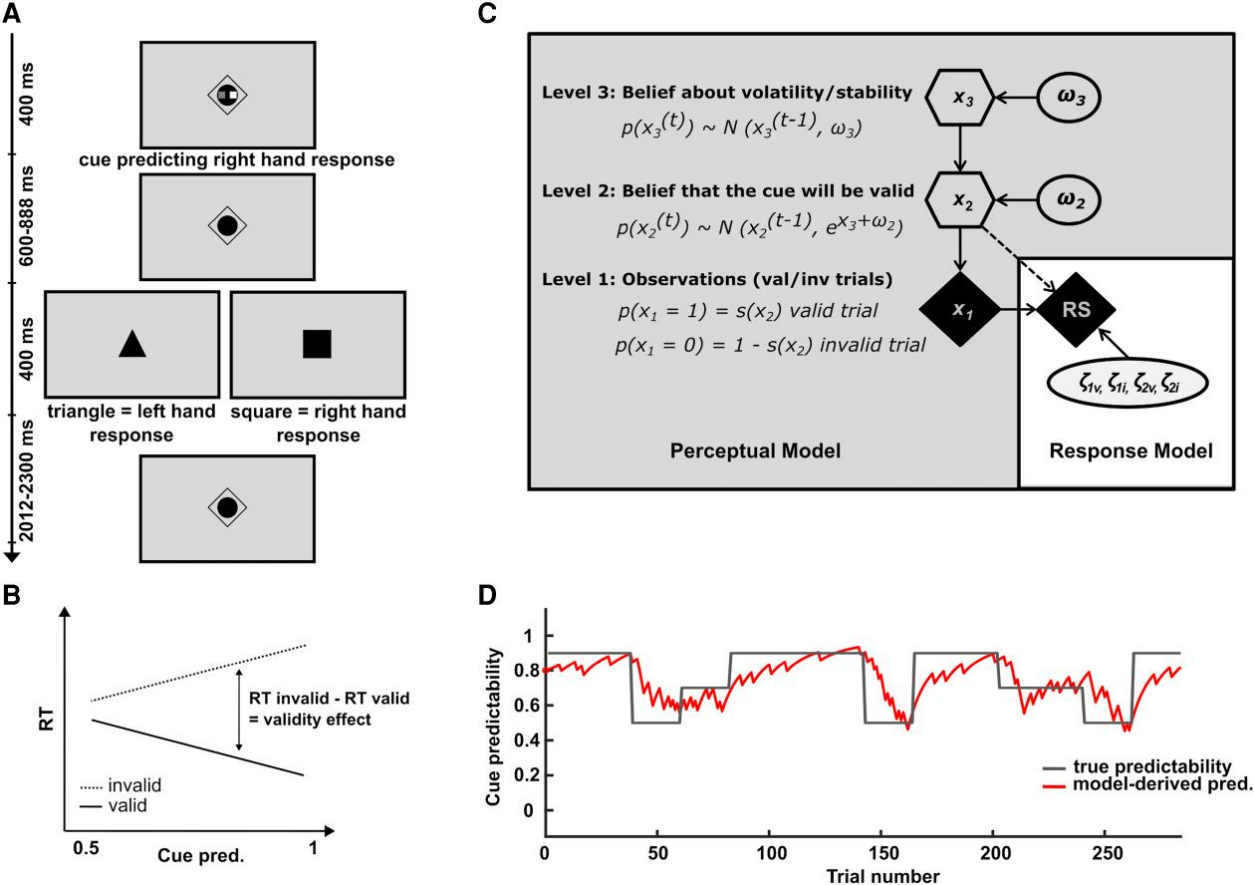
**Experimental paradigm.** (**A**) Within-trial event sequence. A motor cue (white square within the cue stimulus) predicted the hand required for the response, with variable levels of cue predictability. Targets consisted of a triangle or a square requiring a left- or right-hand response (counterbalanced). (**B**) Expected differential effects of cue predictability on RTs in valid and invalid trials. RTs are expected to decrease with increasing cue predictability for valid trials, thereby increasing the cue validity effect. (**C**) The hierarchical Bayesian model for belief updating. The perceptual model (shown on the darker background) incorporates the three states (*x*_1_, *x*_2_ and *x*_3_). Higher levels are influenced by constant parameters *ω*_2_ and *ω*_3_, which affect trial-wise changes on the respective level. Whereas the variables shown in diamonds and hexagons are quantities evolving with time (trials), circled variables are constants. Additionally, the quantities in the hexagons rely upon their previous states in a Markovian fashion. (**D**) Example of one control participant’s trial-by-trial changes in cue predictability ${\hat{\mu }}_{1}^{\left(t\right)}$ (i.e. the participants’ belief that the cue will be valid) in relation to the experimentally manipulated cue predictability over the 284 trials.

Participants performed a computer-based motor cueing version of the Posner paradigm, adapted from Kuhns *et al*.^35^ with modified target objects and central target location (Fig. 1A). The task was administered using Presentation (Version 24, Neurobehavioral Systems Inc., Berkeley, CA, USA) and presented on a 24-inch screen (resolution in pixels 1920 × 1080, 60 Hz sampling rate). A button box (RB-840, Cedrus, San Pedro, CA, USA) collected the participants’ responses.

On each trial, a cue stimulus was shown for 400 ms, consisting of a white and a dark grey square appearing within the fixation diamond. The white square on the left or right side within the cue stimulus indicated a probable left- or right-hand response to the target. After a jittered stimulus onset asynchrony of 1000–1288 ms, a target stimulus appeared for 400 ms, consisting of either a square or a triangle. Participants were asked to respond to the target shapes by pressing a button with either their left or their right hand. The target–response mapping was counterbalanced across participants. The intertrial interval was jittered between 2012 and 2300 ms. All stimuli were presented centrally on the screen.

We experimentally manipulated the cue predictability by introducing changes between ∼50, ∼70 and ∼90% of valid cues (Fig. 1B and D). Participants were unaware of the different levels of cue predictability or when they would change; they were only informed that variations would occur over the course of the experiment. Each participant was presented with the same sequence of trials. Using constant trial sequences is a standard procedure in computational studies of learning processes that require inference on conditional probabilities in time series. Participants performed a total of 284 experimental trials. A break was introduced halfway through the task; the length of this break was self-determined by each participant. The total duration of the task was ∼20 min. To familiarize the participants with the task, we included a practice session in the experiment consisting of 54 experimental trials at a constant 80% cue predictability.

### EEG recordings

EEG was recorded from 63 electrodes (actiCAP, Brain Products GmbH, Gilching, Germany) with a sampling rate of 1000 Hz. All processing steps were performed with EEGLAB^45^ and custom MATLAB scripts (The MathWorks Inc., Natick, MA, USA). Data were filtered with a zero-phase finite impulse response filter (0.5–45 Hz) and down-sampled to 500 Hz. Cue-locked epochs (−1000–4500 ms) were created for artefact correction. Epochs were removed if 5 SD of the joint data probability was exceeded (TS, 11.06 ± 4.97; controls, 15.9 ± 4.85). All data were average referenced and submitted to independent component analysis. Artefactual components were automatically removed by the fully automated multiple artifact rejection algorithm.^46^ Target-locked epochs (−200–800 ms) were created and baseline corrected to the 200 ms pre-cue interval. Topographies were created with the plasma colormap from Matplotlib.^47^

### Behavioural data analysis

Behavioural data analysis of responses and RTs was performed using Spyder (version 5, Python 3.7; https://www.spyder-ide.org). Statistical analyses were performed using JASP (version 0.16, JASP Team^48^). Incorrect and missed trials were excluded from the RT analysis. Three participants had accuracy scores below 2 SD from the overall sample mean (one control participant, 83%; two TS participants, 80.3 and 77.8%). Nevertheless, they were retained in all behavioural and EEG analyses. Accuracy scores (expressed in % of correct responses) and mean RTs were analysed in two separate mixed analyses of variance (ANOVAs) with ‘group’ (TS and controls) as the between-subject factor and ‘validity’ (cue valid and cue invalid) and ‘cue predictability’ (50, 70 and 90% valid cues) as the within-subject factors. In the control group, we expected decreased RTs for valid trials and increased RTs for invalid trials with higher cue predictability. Due to the assumed inverted linear relationship between validity and cue predictability, we expected to find a significant interaction between these two factors. The Greenhouse–Geisser correction of degrees of freedom was applied when the Mauchly test signalled violation of the sphericity assumption.

### Bayesian modelling of trial-wise belief updating

For computational modelling, trial-wise RT was transformed into RS = 1/RT. Differences in motor control in volatile environments between TS and control participants were investigated using a Bayesian observer (HGF) model of trial-by-trial beliefs about cue predictability (i.e. the probability of a valid cue for a given trial). On the basis of each participant’s single-trial RS, this model provides parameters that quantify the individual inference of cue predictability (*ω*_2_) and high-level volatility (*ω*_3_).^37,39^

The model was fitted separately for each participant and consists of two main components: a perceptual (learning) model and a response (observation) model (Fig. 1C). The perceptual model specifies how probability estimates are updated based on observed cue–target associations, while the response model maps these probability beliefs to observable behaviour (i.e. RS). A detailed derivation of the perceptual HGF model equations can be found in Mathys *et al.*^37^

Here, we only summarize the main model characteristics. The perceptual model is composed of Gaussian random walks, which are coupled hierarchically to allow flexible updating of trial-wise beliefs about cue predictability in relation to beliefs about environmental volatility and participant-specific parameters. It contains three hierarchical states (*x*). The first state $\;x_{1}^{\left(t\right)}\;$ describes the binary state in trial *t*, indicating whether the cue was valid ($x_{1}^{\left(t\right)}=1$) or invalid ($x_{1}^{\left(t\right)}=0$). The probability distribution of a valid trial follows a Bernoulli distribution determined by the second state $x_{2}^{\left(t\right)}$: through a sigmoid (softmax) transformation, $x_{2}^{\left(t\right)}$ defines the probability of $x_{1}^{\left(t\right)}$ being valid (1) or invalid (0). $x_{2}^{\left(t\right)}$ evolves from trial to trial as a Gaussian random walk, with its rate of change being modulated by both the third state $x_{3}^{\left(t\right)}$ (the top level of the hierarchy) and a participant-specific updating parameter *ω*_2_. The third state $x_{3}^{\left(t\right)}$ encodes beliefs about the stability (volatility) of cue predictability. Its trial-wise changes are also described as a Gaussian random walk, with a step size being dependent on the participant-specific parameter *ω*_3._

To infer the participant-specific trial-by-trial beliefs about cue predictability and volatility from the RS data, the perceptual model must be inverted. This yields posterior distributions over the three states $x^{\left(t\right)}$. The means of these distributions are denoted by $\mu^{\left(t\right)}$. Variational model inversion using a mean field approximation results in simple analytical update equations, according to which belief updating is driven by weighted prediction errors in an individualized approximate Bayes-optimal scheme (i.e. in relation to each participant’s *ω*_2_ and *ω*_3_ values).^37^ Thereby, the model yields a participant’s trial-wise estimate of the probability that the target will require the cued motor response.

The response model specifies how these estimates translate into observed RS. Following prior work,^35,49^ we assumed that RS depends linearly on the estimated cue predictability prior to observing the trial outcome ${\hat{\mu }}_{1}^{\left(t\right)}$. This estimate of ${\hat{\mu }}_{1}^{\left(t\right)}$ is obtained by a sigmoid transformation of $\mu_{2}^{\left(t-1\right)}$ from the preceding trial. In valid trials, a high probability estimate leads to higher RS (faster responses), while the opposite pattern is expected in invalid trials, with higher cue predictability estimates resulting in lower RS (slower responses). The response model includes the parameters $\zeta_{1}$ (intercept) and $\zeta_{2}$ (slope), which govern the linear relationship in valid and invalid trials, respectively:


$$RS^{\left(t\right)}=\{\begin{array}{c} \zeta_{1v}+\zeta_{2v}{\hat{\mu }}_{1}^{\left(t\right)}\;\;\;\;\mathrm{for}\;x_{1}^{\left(t\right)}\;=\;1\;(\mathrm{valid}\;\mathrm{trials})\\ \;\zeta_{1i}+\zeta_{2i}(1-{\hat{\mu }}_{1}^{\left(t\right)})\;\mathrm{for}\;x_{1}^{\left(t\right)}\;=0\;(\mathrm{invalid}\;\mathrm{trials}) \end{array}$$

We estimated the parameters of the perceptual model (*ω*_2_ and *ω*_3_) and response model ($\zeta_{1}$ and $\;\zeta_{2}$) via variational Bayesian estimation, using the HGF toolbox (version 7.1; Frässle *et al*.^50^; https://www.translationalneuromodeling.org/tapas), implemented in MATLAB (R2023a).

For this study, the key parameters were *ω*_2_ and *ω*_3_ (reflecting the individual learning about cue predictability and volatility) and $\zeta_{2}$ for valid and invalid trials (quantifying how changes in estimated cue predictability lead to changes in RS). These parameters were compared between TS and control participants using independent-samples *t*-tests. The intercepts of the response model equations $\zeta_{1}$ were not analysed further.

### Statistical analysis

All statistical analyses were performed with MATLAB 2021a. For each participant, single-trial EEG data from electrodes FCz (P3a) and CPz (P3b) were regressed against model values at each time point; the regression models (general linear model) included the trial-wise validity (valid/invalid), the HGF model-derived cue predictability ${\hat{\mu }}_{1}^{\left(t\right)}$ and an interaction term (validity × cue predictability). Subsequent regression weights were then averaged for each ERP (P3a amplitude, P3b onset and P3b amplitude). The temporal location and duration of each measurement window (P3a amplitude, P3b onset and P3b amplitude) were determined by grand averages across all conditions and participants. For the P3a amplitude, the measurement window (±40 ms) was centred on the grand average peak latency at 358 ms. For the P3b, a measurement window of 200–300 ms was chosen to capture P3b slope latency differences, and a measurement window of 400–600 ms was chosen to capture amplitude modulations. We want to point out that ‘P3b onset’ is a descriptive term referring to early P3b slope modulations, which have been shown to reflect the P3b build-up, rather than a measure of peak latency.^30^ Regression weights (*b*-values) were then averaged for each measurement window for further statistical comparisons.

At the group level, the averaged regression weights were then submitted to two-tailed one-sample *t*-tests in order to determine significance across the two experimental groups. Two-tailed two-sample *t*-tests were performed to determine significant between-group differences. Additionally, we explored possible relationships between *b*-values and clinical scales to characterize TS symptoms, the YGTSS and PUTS, using Spearman’s rank correlations. Statistical tests with *P* < 0.05 were considered significant.

## Results

### Behavioural results

**Figure 2:**
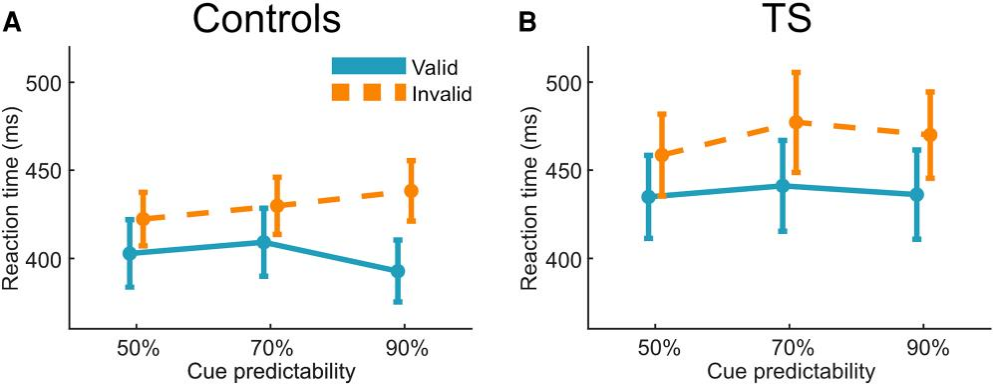
**Reaction times.** RTs for validly and invalidly cued trials in each experimentally manipulated cue predictability level for control participants (**A**) (*n* = 30) and TS participants (**B**) (*n* = 30). A cue predictability × validity × group interaction (ANOVA, *F*_2,116_ = 3.22; *P* = 0.043; $\eta_{p}^{2}$ = 0.053) indicated a differential modulation of cueing effects by cue predictability for TS and control participants.

The mean RT was 404 ms (±95 ms standard deviations) for the control participants and 444 ms (±134 ms) for the TS participants. The mixed ANOVA on RTs revealed significant main effects of validity (*F*_1,58_ = 22.87; *P* < 0.001; $\eta_{p}^{2}$ = 0.283) and cue predictability (*F*_2,116_ = 5.07; *P* = 0.008; $\eta_{p}^{2}$ = 0.08) and a significant cue predictability × validity interaction (*F*_2,116_ = 5.8; *P* = 0.004; $\eta_{p}^{2}$ = 0.091). Responses were slower for invalidly cued targets, and this cueing effect increased with increasing cue predictability. Furthermore, a significant three-way interaction of cue predictability × validity × group (*F*_2,116_ = 3.22; *P* = 0.043; $\eta_{p}^{2}$ = 0.053; see Fig. 2) indicated a differential modulation of cueing effects by cue predictability for TS and control participants. To better interpret this interaction, we separately analysed TS and control groups in two within-subject ANOVAs with factors validity and cue predictability. Main effects for validity were significant for the control group (*F*_1,29_ = 20.2; *P* < 0.001; $\eta_{p}^{2}$ = 0.412) and the TS group (*F*_1,29_ = 8.3; *P* = 0.007; $\eta_{p}^{2}$ = 0.224). Importantly, only the control group showed a significant cue predictability × validity interaction (*F*_2,44.12_ = 9.15; *P* = 0.001; $\eta_{p}^{2}$ = 0.24) that indicated an impact of cue predictability on the cue validity effect.

The mean accuracy was 96.5% (±3.4 SD) for the control group and 94.4% (±5.6 SD) for the TS group (Supplementary Fig. 2). The mixed ANOVA on accuracy revealed significant main effects of validity (*F*_1,58_ = 25.27; *P* < 0.001; $\eta_{p}^{2}$ = 0.304) and cue predictability (*F*_1.67,96.77_ = 3.75; *P* = 0.034; $\eta_{p}^{2}$ = 0.061). Similar to the RT modulation, a significant cue predictability × validity interaction (*F*_2,116_ = 3.99; *P* = 0.021; $\eta_{p}^{2}$ = 0.064) indicated that predictability also modulated the cueing effect for accuracy. All additional results are reported in Supplementary Table 1.

Independent-samples *t*-tests did not reveal any significant differences at the group level in any of the relevant modelling parameters (*ω*_2_, *ω*_3_, ζ_2*v*_ and ζ_2*i*_) between TS and control participants (all *P* > 0.12). Supplementary Fig. 1 shows the observed and predicted (simulated) RS modulations by inferred cue predictability in TS and control participants.

### EEG results

**Figure 3:**
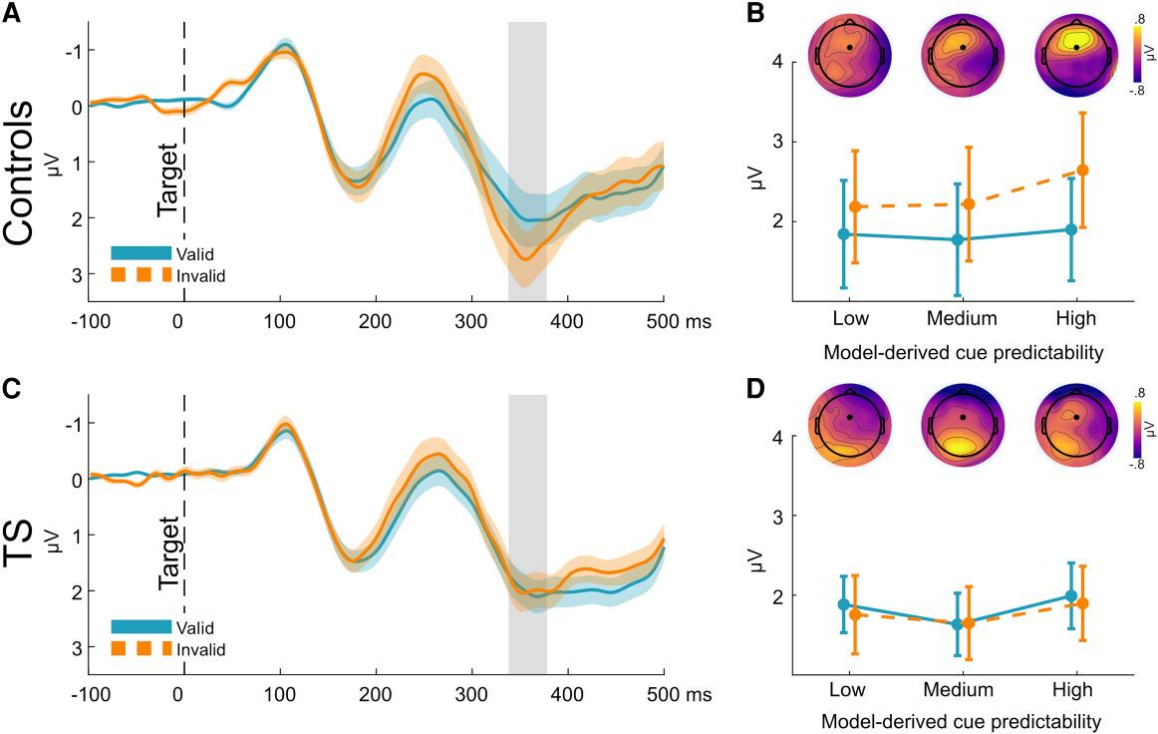
**P3a amplitude modulation.** (**A**) P3a time course for control participants (*n* = 30) at electrode FCz. The shaded vertical bar shows the time window for statistical analyses. (**B**) Topographies showing invalid–valid differences for the control participants. The interaction plot shows P3a amplitudes for valid and invalid trials in three equally sized bins derived from individual participants’ model-derived cue predictability values (low/medium/high). A dot marks electrode FCz. (**C**) P3a time course for TS participants (*n* = 30) at electrode FCz. The shaded vertical bar shows the time window for statistical analyses. (**D**) Topographies showing invalid–valid differences for the TS participants. The interaction plot shows P3a amplitudes for valid and invalid trials in three equally sized bins derived from individual participants’ model-derived cue predictability values (low/medium/high). P3a modulation by validity was increased for control compared to TS participants (two-sample *t*-test, *t*_58_ = −2.05; *P* = 0.04). A dot marks electrode FCz.

P3a amplitude increased with higher model-derived cue predictability (*t*_59_ = 2.06; *P* = 0.04). Moreover, between-group comparisons showed an increased P3a modulation by validity (invalid > valid; see Fig. 3A and C) for control compared to TS participants (*t*_58_ = −2.05, *P* = 0.04; see Fig. 3B and D).

**Figure 4:**
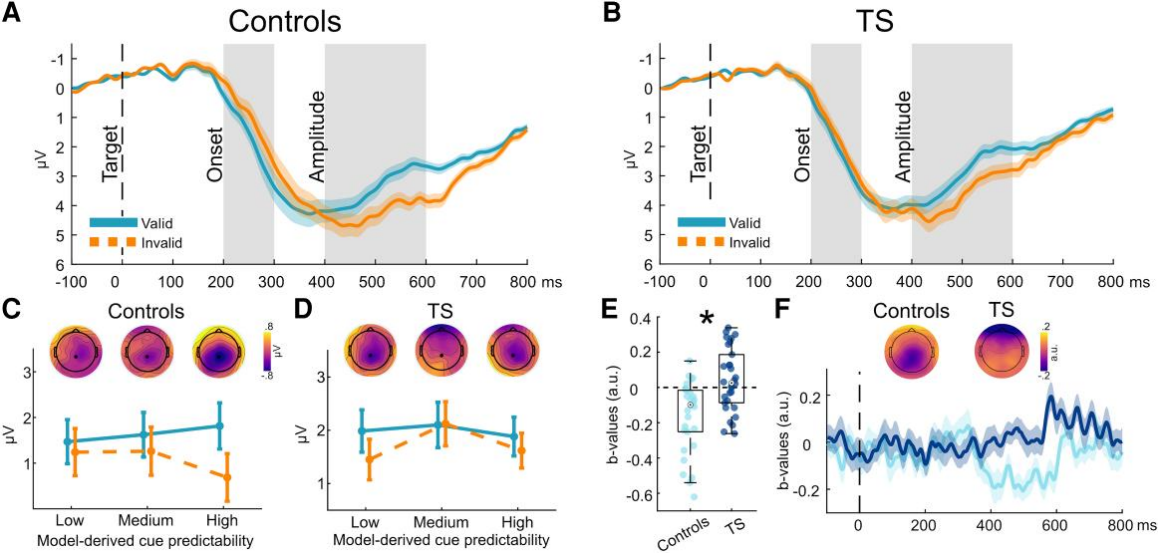
**P3b onset and amplitude modulations.** (**A** and **B**) P3b time course for control (**A**) (*n* = 30) and TS participants (**B**) (*n* = 30) at electrode CPz. Vertical shaded bars show time windows for P3b onset (200–300 ms) and P3b amplitude (400–600 ms) used for statistical analyses. (**C** and **D**) The interaction plots show P3b onset modulations (smaller P3b slope amplitudes correspond to earlier onset, whereas larger P3b slope amplitudes correspond to later onset) for control (**C**) and TS participants (**D**) in three equally sized bins derived from individual participants’ model-derived cue predictability values (low/medium/high). A validity × cue predictability interaction (one-sample *t*-test, *t*_59_ = −2.20; *P* = 0.03) indicated increasingly faster P3b onset for expected valid trials and increasingly slower P3b onset for unexpected invalid trials. Topographies show invalid–valid differences for P3b onset. (**E**) Group differences between model-derived cue predictability *b*-values in the P3b amplitude time window between control and TS participants (two-sample *t*-test, *t*_58_ = 4.16; *P* < 0.001, as indicated by the asterisk). Dots indicate individual participants’ mean values. (**F**) Time course of cue predictability *b*-values at electrode CPz, showing decreased P3b amplitude with higher cue predictability in control but not in TS participants. Topographies were averaged over the P3b amplitude time window. A dot marks electrode CPz.

P3b onset (see Fig. 4A and B) was modulated by validity with delayed onset for invalid compared to valid trials (*t*_59_ = −5.50; *P* < 0.001). A significant validity × cue predictability interaction (*t*_59_ = −2.20; *P* = 0.03) indicated increasingly faster P3b onset for expected valid trials and increasingly slower P3b onset for unexpected invalid trials (see Fig. 4C and D).

P3b amplitude was modulated by validity with increased amplitudes for invalid compared to valid trials (*t*_59_ = 5.10; *P* < 0.001). Amplitudes decreased when cue predictability was high (*t*_59_ = −2.19; *P* = 0.03). The latter effect was diminished in TS participants, leading to a significant between-group difference (*t*_58_ = 4.16; *P* < 0.001) (see Fig. 4E and F). All additional results are reported in Supplementary Table 2.

### Correlations of TS participants’ regression weights with clinical scores

P3a amplitude modulation by cue predictability was negatively correlated with YGTSS-Tic scores (*r* = −0.43; *P* = 0.01). P3b amplitude modulations were not significantly correlated with YGTSS or PUTS scores (all *P* > 0.16). All additional results are reported in Supplementary Table 3.

## Discussion

In this study, we investigated whether sensorimotor predictive processes are altered in individuals with TS. Our findings indicate that increased cue predictability enhances motor preparation in control participants, resulting in faster responses in valid trials and slower responses in invalid trials. Notably, this effect was attenuated in TS participants. At the neural level, we observed diminished P3a validity and P3b cue predictability modulations in TS participants. These results suggest an altered adaptation to sensorimotor predictions in TS.

### Impaired sensorimotor predictions in TS

Typically, responses following valid cues are faster than those following invalid cues, and this difference is amplified with higher predictability of the cue. As in control participants, responses were generally slower for invalid trials in TS participants, suggesting that TS participants did not ignore the cue. However, RTs were not affected by varying cue predictabilities, indicating a deficit in adapting motor preparation to changes of the probabilistic context. Our findings align with prior studies that report difficulties for TS participants in adapting their choices to different levels of probability in probabilistic reinforcement learning tasks.^15-17^ This can be explained by the predictive coding framework as aberrant (precision weighting of) prediction errors to inform top-down predictions about cue–target associations. Within this framework, our observation of impaired processing of sensorimotor predictions aligns with a proposed model for the phenomenology of TS, where tics act as a dysfunctional fulfilment of sensorimotor prediction errors.^51^ Complementarily, the event coding account suggests that stimulus–response features are more strongly bound in TS, a mechanism proposed to underlie the relationship between sensory urges and motor tics.^18,19^ The increased binding of stimulus–response features may hinder the adaptive integration of information provided by the cue, providing an alternative explanation for diminished sensitivity to cue predictability observed in the TS group. Future studies may directly compare competing predictions of both theoretical accounts; however, the present study is not well suited to address this.

Furthermore, similar to RTs, we found that accuracy was modulated by validity, cue predictability and their interaction. This contrasts with earlier findings, where accuracy was modulated exclusively by validity.^35,49^ One possible explanation is the enhanced statistical power provided by the increased sample size in this study. It is also possible that the observed effects are partly due to task modifications implemented to optimize the paradigm for EEG recording. Overall, our results support the idea of impaired sensorimotor predictions in TS.

In contrast to the differences in RT induced by cueing effects, the comparison of the HGF model parameters did not yield any significant differences between TS and control participants. Hence, one could speculate that the exact origin of the attenuated cue predictability modulation may differ between TS participants. For instance, some TS participants may show slower inference of cue predictability *per se* (as reflected in lower *ω*_2_ parameters of the perceptual model), whereas others may show an aberrant regulation of RS to inferred cue predictability ($\zeta_{2}$ parameters of the response model). While the present study does not allow for analyses of different participant clusters or subgroups, our approach could be used in future large-scale behavioural studies for a more fine-grained model-based characterization of (mal)adaptation to sensorimotor predictions.

### Neural activity during sensorimotor processes in TS

We found increased P3a amplitudes with increased cue predictability, likely indicating greater attention allocation in predictable compared to random environments. While there was no between-group difference, this modulation was negatively correlated with tic symptomatology in the TS group, suggesting that severely affected individuals exhibited less adaptation of P3a amplitudes to changes in cue predictability. Additionally, we observed increased P3a amplitudes following invalid cues for the control participants, consistent with previous findings.^52,53^ This modulation was diminished in TS participants, suggesting a decreased allocation of attention towards stimuli requiring behavioural adaptation (in our case, the reprogramming of a prepared motor response). This finding aligns with previous reports of attenuated P3a amplitudes in TS during reinforcement learning and stop-signal tasks.^17,54^ Importantly, our results cannot be explained by deficits in orienting or re-orienting of spatial attention since the targets appeared at the centre of the screen. Together, our findings suggest decreased modulation of P3a amplitudes in TS participants in response to events that signal the need for behavioural adjustments.

Furthermore, we observed a delayed P3b onset for invalid compared to valid trials, with this difference increasing with higher cue predictability. This suggests that P3b onset serves as a marker of motor response preparation, mirroring the behavioural RT adaptation to increasing cue probabilities. The P3b has been conceptualized as being involved in, or possibly bridging, stimulus categorization and response preparation.^29^ Further, it has been suggested that the P3b constitutes a build-to-threshold variable, with its timing scaling with RT when stimulus categorization is required.^30-32^ Our study supports and extends these findings by showing that cue predictability impacts P3b onset latencies, suggesting a functional relationship with behavioural adaptation in changing predictive environments.

Finally, P3b amplitudes were greater in invalid trials than in valid trials, reflecting their association with behavioural relevance and their inverse relationship with frequency.^29^ This fundamental property of P3b amplitude modulation was comparable between both groups. Moreover, P3b amplitudes have been shown to positively predict learning and subsequent behavioural adaptation to task demands.^55,56^ We observed reduced P3b amplitudes with increasing cue predictability (i.e. in more predictable environments) in control participants, aligning with the notion that less updating—or a reduced Bayesian surprise signal—is required when the internal model of the (predictable) environment is appropriately updated.^57-59^ Conversely, elevated P3b amplitudes in more predictable environments, as observed in a subset of participants in the TS group, may suggest insufficient internal model formation, necessitating ongoing updating and, therefore, reflecting an attempt to establish a model in a context where cue–target relationships are no longer random. Given the proposed relationship between P3b modulations and the updating of cue–target mapping, the observed group difference may also be relevant to the attenuated behavioural adaptation in TS participants.

We must acknowledge several limitations of our study. First, the limited sample size for between-group comparisons may affect the generalizability and statistical power of our results. Future studies with larger cohorts are needed to validate our findings and provide more robust conclusions. Second, we did not account for comorbid disorders in our analyses. Many individuals with TS have comorbid disorders such as attention-deficit/hyperactivity disorder or obsessive-compulsive disorder, which can influence both neurophysiological measures and behavioural performance. Third, since responses fall in the measurement window of the P3b amplitude, we cannot rule out an influence of intra-individual variability in evoked EEG components. However, since we observed comparable P3b amplitudes between control and TS participants (controls, 3.6 ± 1.97; TS, 3.12 ± 1.96; *t*_58_ = 0.98; *P* = 0.33), we remain confident that intra-individual variability was not the primary source of the between-group differences. Fourth, it is worth noting that 17 out of 30 TS participants were using medications commonly prescribed to alleviate TS symptoms. These medications affect the dopaminergic, serotonergic and cannabinoid systems and may have influenced task performance. Therefore, the observed between-group effects should be interpreted with caution, as they may partially reflect the impact of pharmacological treatments.

To summarize, we observed distinctive behavioural and neural patterns in TS participants that indicate impairments in adapting their behaviour to a probabilistic context. We hope that the insights gained contribute to a more nuanced understanding of the sensorimotor deficits in TS and help refine hypotheses to advance our understanding of this multifaceted disorder further.

## Supplementary Material

fcaf458_Supplementary_Data

## Data Availability

All code is publicly available (https://osf.io/xy5kw). Data can be made available conditionally to data sharing agreements signed by the patients within the legal framework of the General Data Protection Regulation of the European Union.
