# Supplementary material for: Alterations of sensorimotor predictive processes and their electrophysiological signatures in Tourette syndrome
Source: Brain Commun. 2025 Nov 21;7(6):fcaf458. doi: 10.1093/braincomms/fcaf458 (PMC12699222; doi:10.1093/braincomms/fcaf458)
Supplement: fcaf458_Supplementary_Data [file fcaf458_supplementary_data.pdf]

Supplementary Table 1: Results of mixed ANOVAs for accuracy and reaction times.

	df	F-value	p	eta^2p
Accuracy				
Validity	1,58	25.2	**< 0 .001**	0.304
Validity x group	1,58	1.4	0.23	0.024
Cue predictability	2,116	3.7	**0.02**	0.061
Cue predictability x group	2,116	1.6	0.20	0.027
Validity x cue predictability	2,116	3.9	**0.02**	0.064
Validity x cue predictability x group	2,116	1.9	0.20	0.033
Group	1,58	1.1	0.28	0.020
Reaction times				
Validity	1,58	22.8	**< 0.001**	0.283
Validity x group	1,58	.04	0.83	< 0.001
Cue predictability	2,116	5.0	**0.008**	0.08
Cue predictability x group	2,116	.41	0.66	0.007
Validity x cue predictability	2,116	5.8	**0.004**	0.091
Validity x cue predictability x group	2,116	3.2	**0.04**	0.053
Group	1,58	1.5	0.21	0.026
TS participants – reaction times				
Validity	1,29	8.3	0.007	0.224
Cue predictability	2,58	2.6	0.07	0.085
Validity x cue predictability	2,58	1.2	0.28	0.042
Control participants – reaction times				
Validity	1,29	20.2	< 0.001	0.412
Cue predictability	2,58	2.9	0.06	0.091
Validity x cue predictability	2,58	9.1	< 0.001	0.240

Supplementary Table 2. Within- and between-group analyses of regression weights.

One-sample t-tests	df	t-value	p
P3a			
Validity	59	1.28	0.20
Cue predictability	59	2.06	**0.04**
Validity x cue predictability	59	0.10	0.44
P3b onset			
Validity	59	-5.50	**< 0.001**

Cue predictability	59	-0.46	0.83
Validity x cue predictability	59	-2.20	**0.03**
P3b amplitude			
Validity	59	5.10	**< 0.001**
Cue predictability	59	-2.19	**0.03**
Validity x cue predictability	59	1.18	0.24

Two-sample t-tests	df	t-value	p
P3a			
Validity	58	2.05	**0.04**
Cue predictability	58	0.48	0.62
Validity x cue predictability	58	0.31	0.75
P3b onset			
Validity	58	-1.78	0.07
Cue predictability	58	0.34	0.73
Validity x cue predictability	58	-1.82	0.07
P3b amplitude			
Validity	58	0.42	0.67
Cue predictability	58	-4.16	**< 0.001**
Validity x cue predictability	58	0.36	0.71

Supplementary Table 3: Spearman correlations of regression weights with clinical scores.

	YGTSS Tic Score		PUTS	
P3a regression weights	r	p	r	p
Validity	0.01	0.93	0.01	0.92
Cue predictability	**-0.43**	**0.01**	-0.06	0.73
Validity x cue predictability	0.20	0.20	0.29	0.11
P3b onset regression weights				
Validity	-0.13	0.48	0.01	0.94
Cue predictability	-0.08	0.65	-0.01	0.96
Validity x cue predictability	0.12	0.51	**0.35**	**0.05**
P3b amplitude regression weights				
Validity	0.11	0.55	0.26	0.16
Cue predictability	0.19	0.29	0.08	0.66
Validity x cue predictability	0.16	0.39	0.13	0.46

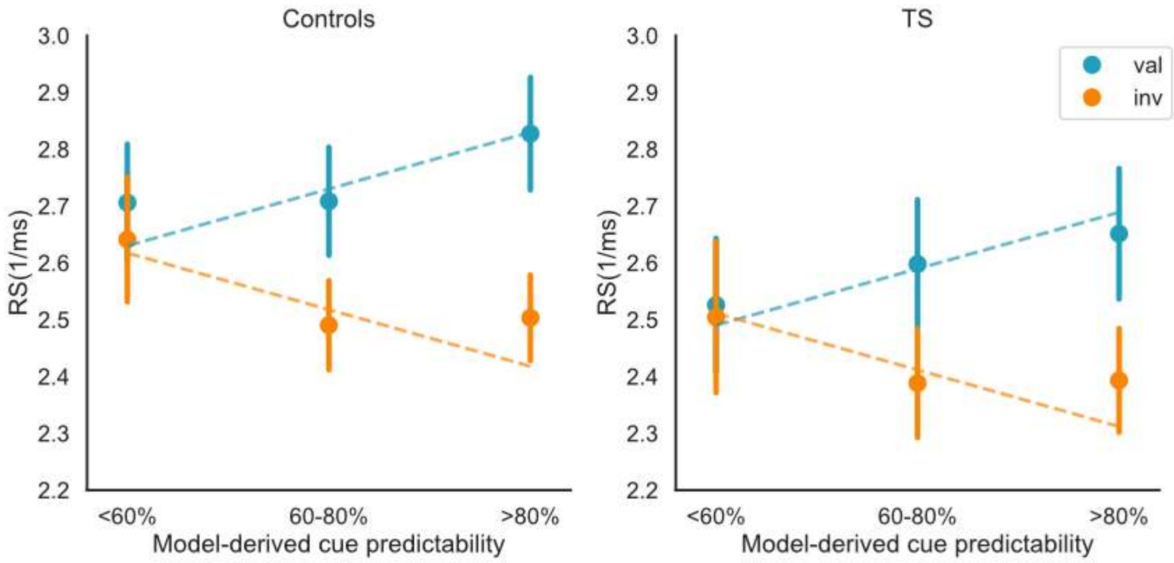

Supplementary Figure 1. Modeling results. Observed and predicted mean response speed (RS = 1/reaction time) for valid and invalid trials in relation to the participants' trial-by-trial estimates of the cue predictability $\hat{\mu}_1^{(t)}$ for control participants (A, *n* = 30) and TS participants (B, *n* = 30). Point markers show the average observed response speed data (± SEM), while dashed lines show predicted response speed in the correspondings bins, calculated on the basis of the average response model parameters in each group. Independent-samples t-tests of modelling parameters did not reveal any significant differences between TS and control participants (all p > .12).

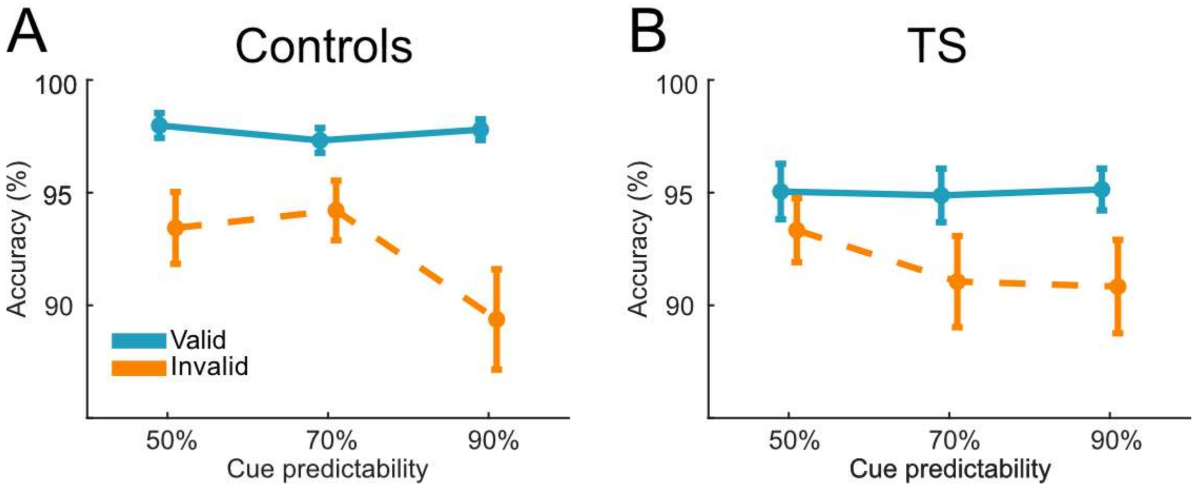

Supplementary Figure 2. Behavioral accuracy. Accuracy scores for validly and invalidly cued trials in each experimentally manipulated cue predictability level for control (A, *n* = 30) and TS participants (B, *n* = 30). Responses were less accurate for invalidly cued targets, and this cueing effect increased with increasing cue predictability, as evidenced by a *cue predictability x validity* interaction (ANOVA, $F_{2,116} = 3.99$, $p = 0.021$, $\eta^2_p = 0.064$).
